# Policy design and implementation: a quantitative evaluation and empirical study of China’s rural sports policy based on the PMC index model

**DOI:** 10.3389/fpubh.2024.1430173

**Published:** 2024-08-29

**Authors:** Baibing Chen, Qizhi Zhu, Dongfang Xie, Yaoxian Lao, Hong Li

**Affiliations:** ^1^School of Public Policy And Management, Guangxi University, Nanning, China; ^2^School of Physical Education, Guangxi University, Nanning, China; ^3^College of Physical Education And Health, Guangxi Science & Technology Normal University, Laibin, China

**Keywords:** rural sports, rural sports policy, policy design and implementation, PMC index modeling, quantitative evaluation

## Abstract

The formulation and implementation of a rural sports policy is an important means of promoting rural sports, improving the physical wellbeing of farmers, and enhancing the cohesion of rural communities. However, introducing such a policy faces problems in the process of specific policy practices, such as poor effective implementation, a lagging implementation effect, and goal cognitive bias. How to look at the current rural sports policy implementation blockage problem and the governance of the blockage, in order to improve the level of rural sports public service, is the focus of this paper’s research. On this basis, this paper selects 56 policy texts, issued from 2002 to 2023, that are highly relevant to rural sports and have high timeliness and authority from the sports policies issued in China. Also, ROST CM6 software is used to count high-frequency words; this study then draws keyword social network mapping for the visual analysis of policy preferences and selects 20 rural sports policy texts as typical samples. Finally, a policy modeling research consistency (PMC) index model is used to evaluate the texts comprehensively and quantitatively. The results show that the overall design of China’s rural sports policies is relatively reasonable. However, the consistency and effectiveness of their implementation need to be improved. Twenty representative policy texts have an average PMC index score of 5.96, with a concave index of 3.04 (which is good overall), with the highest mean value for rural sports policies at the national level. This is followed by the second highest value at the municipal and county levels, and the smallest at the provincial level. Therefore, in the future formulation and implementation of rural sports policies, a multi-dimensional rural sports policy system should be constructed. This would help to strengthen the consistency and effectiveness of the implementation of the policy system and promote the high-quality development of rural sports.

## 1 Introduction

Rural sports are a key engine and play an important role in improving the physical wellbeing of the masses. Sports enhance community cohesion and promote the construction of spiritual civilization in rural areas (1). In short, sports play an irreplaceable role in promoting the development of rural areas in China. However, due to the accelerated process of urbanization, rural population loss is a serious issue, and the phenomenon of left-behind children and the older adults being impoverished and returning to poverty due to illness has surged (2). As a result, the health of the rural population on the road to common prosperity has attracted a lot of attention. The Fifth National Physical Fitness Monitoring Bulletin states that “the physical fitness of young children, adults, and the older adults in urban areas is better than that in rural areas. Also, the difference between urban and rural areas is particularly obvious for males, and the physical functions and physical fitness of adults and the older adults who participate in physical exercise are better than those of the same gender and age group who do not participate in physical exercise. The tendency is, the higher the frequency of physical exercise and the higher the intensity of that exercise is, the better the fitness. In terms of physical health, those who participate in physical exercise have better physical fitness than those who do not participate in physical exercise. In terms of mental health, those who participate in physical exercise have a lower chance of suffering from depression and anxiety than those who do not participate in physical exercise” (3). In addition, the China White Paper reports that, “the higher the level of physical fitness, the lower the percentage of chronic diseases” (4). To sum up, good physical fitness provides the basis and guarantee for a high level of health. In turn, good health reduces medical expenditures, eases the pressure on social security, promotes social stability and harmony, and lays a solid foundation for achieving the goal of common prosperity.

Rural sports are part of the focus of national fitness but are also the weakest link of national fitness. This is due to the imbalance between urban and rural sports development, which is directly reflected in the lack of rural sports venues and facilities and farmers’ low awareness of and participation in sports activities (2). The Report on the Sports Status of Urban and Rural Residents in China reveals that the participation rate of urban residents in physical activity is significantly higher than that of rural residents. Specifically, nearly 70% of urban residents participate in weekly physical activity, while less than 30% of rural residents do so (5). This is due to two misconceptions among farmers. One is that engaging in agricultural activities has already ensured physical activity, and therefore, no further fitness activities are required. The other problem is that farmers do not recognize the relationship between physical activity and their own health (6). In reality, in the countryside, the number of people engaged in agricultural activities and the average time spent in those agricultural activities have both been declining continuously. With the rapid advance of urbanization, rural population loss is a serious issue; most of those who stay in the countryside are old people, women, and children (2). China’s rural development report mentions that the proportion of the rural population over 60 years of age is expected to reach 25.3% in 2025, or about 124 million people (7). Also, only a small portion of the young and middle-aged people will actually take part in agricultural production. With the advancement of large-scale agricultural operations, traditional human labor is gradually being replaced by agricultural mechanization, with the mechanization rate of crop cultivation and harvesting exceeding 72% (7), which improves production efficiency. In addition, the time farmers spend participating in farm work is shortening. More and more people are being released from agricultural production, and the physical activities associated with farming are being greatly reduced. In addition, farm work mainly focuses on some specific muscle groups, and this will lead to insufficient or unbalanced development of other muscle groups. Improper posture and repetitive exercise not only fail to improve the physical health of farmers but may also lead to muscle strains, joint pains, and other problems. Therefore, farmers should engage in scientifically-approved physical exercise to enhance their physical fitness, prevent sickness, and help build a healthy China.

Policies are important drivers of development. The development and implementation of rural sport policies can be effective in promoting high quality rural sport development. The key processes of public policy include: decision making, implementation, and evaluation (feedback) (8). Of those processes, designing an idealized policy is a prerequisite for the start of policy implementation (9). Adopting appropriate measures to ensure coherence and consistency in policy implementation and execution is also an important element of the policy process (10). Predicting the effects of a policy will influence subsequent policy revisions and the generation of new policies (11). Idealizing the policy content and design framework at the policy design stage can effectively avoid potential risks arising during policy implementation. However, even the idealization of the policy design process cannot completely avoid the deviations arising from the dynamic process of implementation. Attention must also be paid to the coherence and consistency of the policy implementation process, so that policy implementation and execution can be continuously adapted in the complex and changing environment. Therefore, based on the reality of China, research on the design and implementation of rural sports policies in China is of great significance in promoting the development of rural sports in the new era. Such research is also important in the comprehensive implementation of the national strategies of national fitness, a healthy China and rural revitalization in the new journey. The rest of this article is organized as follows: Section 2 presents the literature review, Section 3 describes the data sources and research methodology, Section 4 presents the results of the study, and the results are analyzed in Section 5. Finally, Section 6 concludes the study and discusses policy recommendations.

## 2 Literature review

Sports, with its inherent humanistic spirit and multiple functions, has an indispensable mission and even a responsibility in rural development. Current academic research on rural sports focuses on the stages of rural sports development, rural sports governance, rural public sports services, and interventions for rural sports activities (12). In terms of rural sports development, there is an imbalance between urban and rural areas and regions in terms of sports venues and facilities. The total supply of venues and facilities in rural areas is insufficient, and the types of sports facilities are relatively homogeneous (13). China’s public sports services are again imbalanced between urban and rural areas. Specifically, rural public sports facilities are out of touch with residents’ sports and fitness needs, and rural residents’ satisfaction and participation with the services are not high (14). In terms of rural physical activity interventions, the fun of the sport itself, the individual’s sense of social group, and the unique social and cultural environment are all factors that influence rural residents’ participation in physical activity (15, 16). China’s rural sports policy promotes the construction of rural sports facilities, talent training, and sports activities through government support and resource investment. Currently, most scholars mainly focus on the means of implementation, interventions, and policy evaluation of rural sport policies. However, there has been virtually no quantitative research on rural sport policy design and implementation. Idealized policy design can be divided into idealized policy content design and idealized policy process design (17). The former involves the formulation of specific content, objectives, and measures of a policy to achieve specific public policy objectives. The latter, on the other hand, is concerned with the methods, procedures, and tools used in the policy development process to ensure that the policy design process is transparent, participatory, and effective. Distinguishing between the content and the process of policy design as a way of ensuring the comprehensiveness and effectiveness of the design process ensures policy resilience, stability, and integrity (18, 19). During the content design process, attention needs to be paid to appropriate design methodologies and design tools to achieve content design rigor and feasibility, such as those related to cultural differences, population size, limited human capital, and other relevant policies that affect the physical health of people living in rural communities (20, 21). In the policy implementation process, individuals, organizations, interventions, and contexts are factors that influence the implementation of rural physical activity policies (22). In terms of policy assessment, rural physical activity policies in China use a higher proportion of supply-based and environment-based tools and a lower proportion of demand-based tools. In addition, synergy between policy subjects must be further strengthened (23). Overall, an idealized policy design framework can improve the results of policy implementation and help achieve policy objectives.

At present, most research on rural sports policy is comprised of theoretical discourse and qualitative analysis, such as policy tool analysis, text analysis, and assessment by university experts or rural grassroots managers (24). However, few studies exist that look at the comprehensive quantitative evaluation of rural sports policy from policy text, and no literature exists on the quantitative evaluation and analysis of a rural sports policy using the PMC index model. Among the quantitative evaluation methods of policies, the most famous is the evaluation of the U.S. New Deal program by sociologist A. S. Stephan, which used the experimental design method. This was followed by OF Poland’s “Three E’s” evaluation classification method, Suchman’s five-category assessment method, and Wollmann’s classic policy evaluation method. The PMC index model is currently a more advanced and objective international policy text evaluation method. This method not only can evaluate the consistency of policies but can also evaluate the specifics of a policy, so as to put forward specific improvement opinions on the policy’s shortcomings in a targeted manner (23). At present, the PMC index model is mostly used by scholars in the fields of public administration, social security and social organizations, scientific research and technical services, etc. In the field of sports, the model has mainly been employed to analyze the sports industry (25, 26) and the sports tourism industry (27), but has not yet been involved in the field of rural sports policy.

Rural sports serve as a crucial catalyst and play a significant role in enhancing the physical well-being of the general population. The formulation and implementation of a rural sports policy is an important means of promoting rural sports, improving the physical wellbeing of farmers, and enhancing the cohesion of rural communities. However, current research on rural sports primarily focuses on the development, governance, public sports services, and promotional functions of rural sports. There is a lack of a robust policy evaluation system, resulting in few studies that have quantitatively analyzed rural sport policies. As a result, the effectiveness of policy implementation remains questionable. Therefore, this study will start with the rural sports policy texts issued by the state, provinces, cities, and counties. In total, 56 rural sports policies will be screened to conduct deep text mining, using ROST CM6 and VOSviewer software to extract high-frequency words and establish the social network mapping of China’s rural sports policies. Then, 20 representative policies are selected (from the 56) to conduct multi-input–output analyses and PMC index calculations. Then, PMC surface and radar charts are constructed from the scores of each index. Finally, the policies are quantitatively evaluated and analyzed, the problems are identified, and corresponding suggestions are made. By developing the PMC index evaluation model, this study offers a novel approach for conducting quantitative research on China’s rural sports policies. It also provides a practical foundation for future revisions, adjustments, and establishment of rural sports policies. Furthermore, it broadens the application scope of the PMC index model.

## 3 Data sources and research methodology

### 3.1 Text mining and text analysis

**Figure 1:**
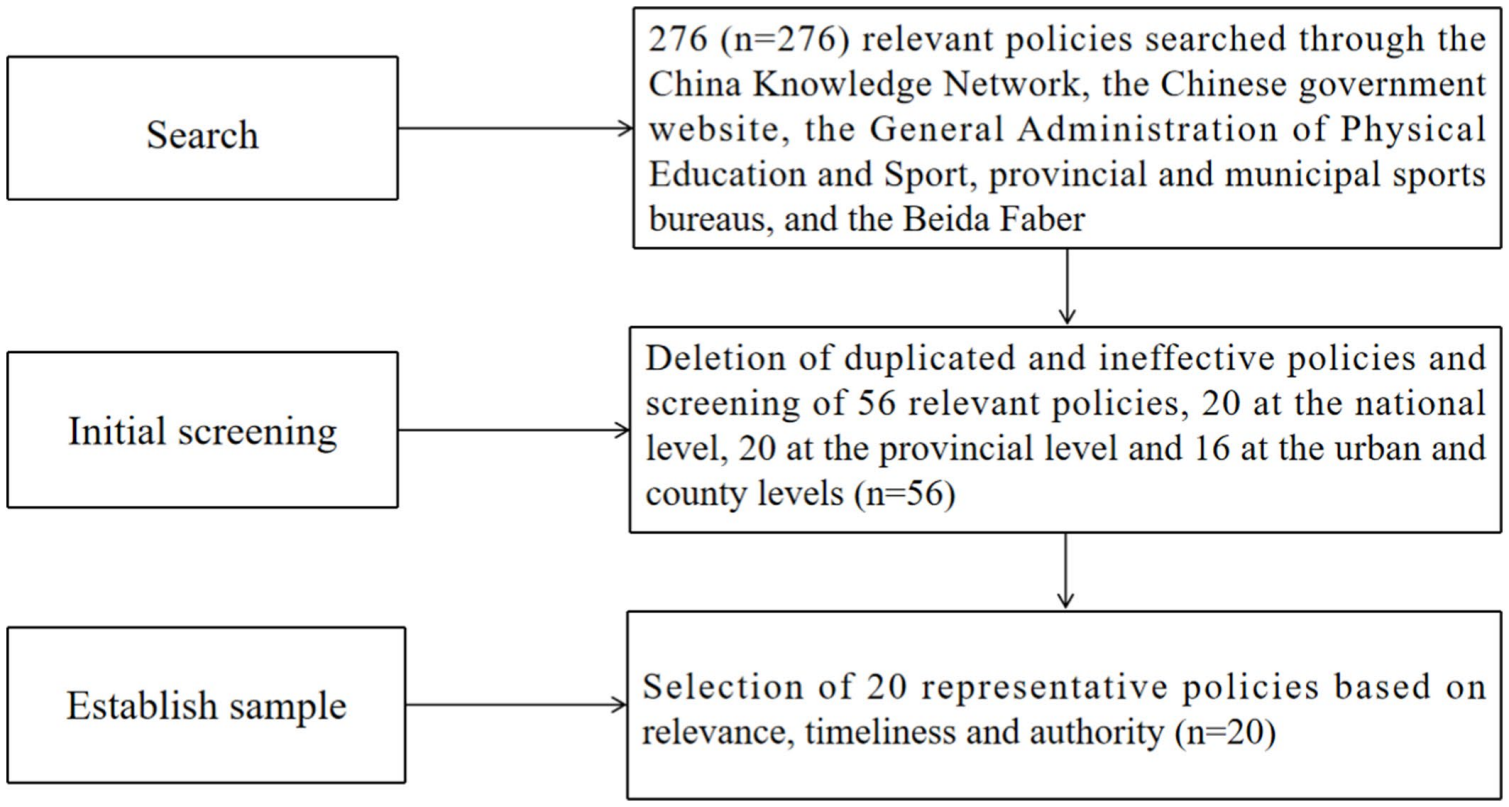
Policy screening flowchart.

**Figure 2:**
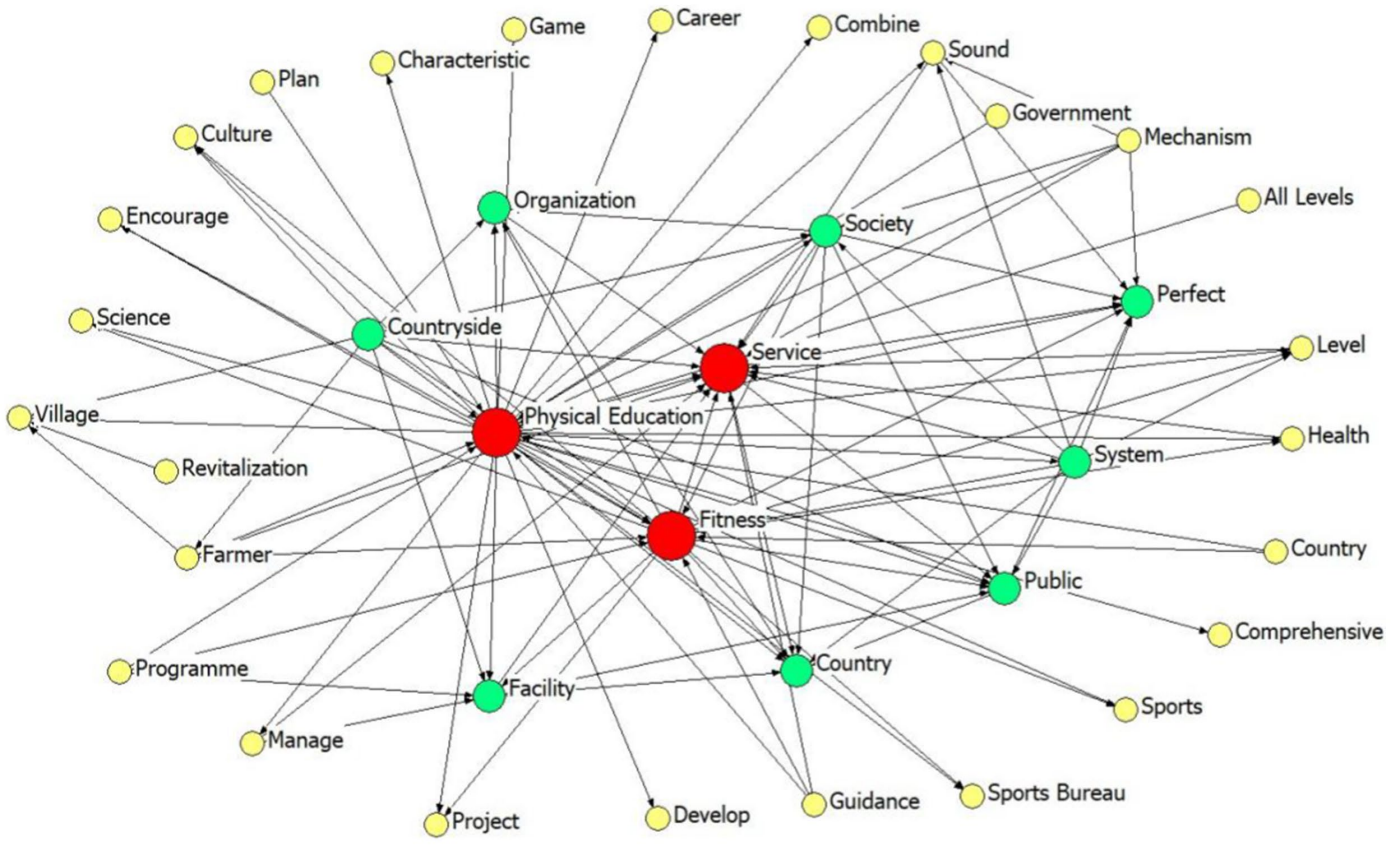
Social network mapping of high-frequency words in China’s rural sports policy texts.

To ensure the accuracy and completeness of the study data, this study will select the samples in accordance with the following steps: (1) By utilizing the keywords “national fitness,” “rural sports,” “farmer sports” and “mass sports,” a total of 276 relevant policy documents were retrieved from the “Peking University Law and Regulation Database,” as well as from the General Administration of Sport of China, provincial and municipal sports bureaus, and local governments. (2) Based on the criteria of completeness, timeliness, and high relevance, excluding incomplete, expired or updated information, that which is unrelated to rural sports or has less connection with rural sports, and invalid policies such as “standard category” and “approval category,” ultimately 56 effective rural sports policies were obtained; (3) Through interviews with the policy makers and executors of the Policy and Regulations Department of the General Administration of Sport of China, the Chinese Farmers’ Sports Association, and some provincial sports bureaus (see Appendix 1 for the interview outline), the research group collated the interview data and conducted two rounds of group discussions to finally determine the 20 representative policies, and the PMC index model was utilized for quantitative analysis and evaluation. The policy screening process is shown in Figure 1, in which 56 policy texts were imported into the system for word frequency statistics using the ROST CM tool. High-frequency words were extracted by eliminating the irrelevant and generic words of this study, which finally resulted in the keyword semantic network diagram of the rural sports policy texts (see Figure 2).

In social network mapping, high-frequency subject terms are combined in the form of a network, and the structural relationship between high-frequency subject terms is visualized in images. The nodes are used to represent the high-frequency theme words; the larger the center node is, the more important the theme word is (28). The lines between the nodes reflect which policies are closely related to each other and which policies are more isolated in the network (29). Figure 2 shows that “Physical Education, Service, Fitness” has the largest node, the strongest degree of centrality, and the most connections with other subject terms, which are the core subject terms of the policy text. This indicates that the main contents of the policy are rural sports, sports public services, national fitness, social sports organizations, etc. The second set of keywords includes “Countryside, Organizations, Society, Facility, Public and Perfect”. It is evident that the current Chinese rural sports policy places emphasis on establishing a rural public sports service system, enhancing the provision of public sports facilities, and improving the service capacity of rural sports social organizations. Another objective is to seamlessly integrate farmers’ health and effectively promote the robust development of rural sports.

### 3.2 Variable selection and parameter setting

The PMC index model requires a comprehensive consideration of all variables throughout the process of policy formulation and implementation, and the role played by certain variables cannot be isolated or ignored (30). Therefore, when establishing indicators for evaluating rural sports policies in China, all relevant and potentially relevant variables should be taken into account. The selection of first-level indicators in this study is based on the classification of variables proposed by Ruiz (2008), Yong’an and Haituo (31), and Xiong et al. (32), etc., and is derived from the high-frequency words of the subject words in the statistics of the policy text as well as the mapping of the semantic analyses. Also, considering the uniqueness of the text of the rural sports policy, a total of nine first-level variables were established, namely: Type of policy (X1), policy level (X2), target audience (X3), policy timeliness (X4), policy content (X5), policy function (X6), policy guarantee (X7), Policy target (X8), Policy evaluation (X9). Secondly, by analyzing the content of the sample policy texts and referring to the selection of secondary indicators of PMC by Zhang Yong’an, Cheng Meichao, Li Lihui, Bu Lingtong and other scholars in the relevant studies, several secondary variables were set under the above nine primary variables, and the complete evaluation index system of rural sports policies in China is presented in Table 1.

**Table 1:** Indicators and secondary variables for evaluating rural sports policies in China.

Level 1 variable	Level 2 variable	Evaluation indicators for secondary variables	Source or basis of variable
X1 Type of policy	X1-1 Anticipate	Whether the policy is predictive: yes 1, no 0	Modification of the study with reference to Yongan Zhang, Meichao Cheng and Ruiz Esurada
	X1-2 Tuition	Whether the policy is instructive: yes 1, no 0	
	X1-3 Plan	Whether the policy is planned or not: yes 1, no 0	
	X1-4 Supervisory	Whether the policy involves regulation: yes 1, no 0	
	X1-5 Suggestion	Whether the policy makes recommendations: yes 1, no 0	
X2 Policy level	X2-1 National level	Whether the policy is issued by a national organ: yes 1, no 0	Reference to research by Li Lihui et al. for modification and population screening
	X2-2 Provincial level	Whether the policy is issued by provincial or municipal authorities: yes 1, no 0	
	X2-3 City-county level	Whether the policy is issued by a city or county agency: yes 1, no 0	
X3 target audience	X3-1 Government office	Whether the object of action involves a government department: 1 for yes, 0 for no	Reference to research modifications and population screening by Hou Tiantian, Cai Dongsong, and others
	X3-2 An institution directly affiliated with an employer	Whether the object of the action involves directly subordinate institutions: yes 1, no 0	
	X3-3 Social organization	Whether the target involves social organizations: yes 1, no 0	
	X3-4 Peasants	Whether the object of action involves farmers: 1 yes, no 0	
X4 Policy timeliness	X4-1 Short-term	Policies with a statute of limitations of 2 years or less: yes 1, no 0	Revised based on the research of Zhou Wei and Cai Lihui
	X4-2 Mid-term	Policy limitation of 2–5 years: yes 1, no 0	
	X4-3 Long term	Policy statute of limitations is more than 5 years: yes 1, no 0	
X5 Policy content	X5-1 Farmers’ sports	Whether the policy involves farmers’ sport: 1 for involvement, 0 for non-involvement	Reference policy texts, theme words, and high-frequency word statistics
	X5-2 Rural sports activities	Whether the policy addresses the content of rural physical activity: 1 being involved, 0 for being not involved	
	X5-3 National fitness	Whether the policy involves fitness for all: 1 for involvement, 0 for non-involvement	
	X5-4 Public sports facilities	Whether the policy involves the content of public sports facilities: 1 for involvement, 0 for non-involvement	
	X5-5 Fitness instructor	Whether the policy addresses the content of the Township Fitness Guidelines: 1 for addressing, 0 for not addressing	
	X5-6 Public service system for national fitness	Whether the policy involves the content of public services for fitness for all: 1 for involvement, 0 for non-involvement	
	X5-7 Policy and institutional safeguards	Whether the policy addresses the content of the policy system safeguards: 1 for addressing, 0 for not addressing	
	X5-8 Sports in the countryside	Whether the policy involves the development of rural sports talents: 1 for involvement, 0 for non-involvement	
	X5-9 Rural sports talent development	Whether the policy involves the construction of rural teacher teams: 1 for involvement, 0 for non-involvement	
X6 Policy function	X6-1 Clear-cut powers and responsibilities	Whether the policy has a clear authority and responsibility function: yes 1, no 0	Revised based on the research of Hu Haoyu, Hu Ruochen, and others
	X6-2 Implement	Whether the policy has a follow-up function: yes 1, no 0	
	X6-3 Normative guidance	Whether the policy has a normative and guiding function: yes 1, no 0	
	X6-4 Institutional constraints	Whether the policy functions as an institutional constraint: yes 1, no 0	
	X6-5 Coordinate and push forward	Whether the policy has an integrative function: yes 1, no 0	
X7 Policy guarantee	X7-1 Government service	Whether the policy has government organizational leadership: yes 1, no 0	Revised based on the research of Hou Tiantian, Shi Lizhen, and others
	X7-2 Financial subsidies	Whether the policy has a financial subsidy, yes 1 no 0	
	X7-3 Talent support	Whether the policy provides talent support: yes 1, no 0	
	X7-4 Laws and regulations	Whether the policy is guaranteed by laws and regulations: yes 1, no 0	
X8 Policy target	X8-1 National fitness	Whether the policy addresses the goal of fitness for all: yes 1, no 0	Reference policy texts and high-frequency word statistics
	X8-2 Health for all	Whether the policy addresses the goal of health for all: yes 1, no 0	
	X8-3 Building a national fitness service system	Whether the policy builds the goal of a national fitness service system: yes 1, no 0	
	X8-4 Equalization of public services in fitness for all	Whether the policy addresses the goal of equalization of public services for all: yes 1, no 0	
	X8-5 Rural rejuvenation	Whether the policy addresses rural revitalization objectives: yes 1, no 0	
	X8-6 Quality development of farmers’ sports	Whether the policy addresses the goal of high-quality development of farmers’ sports: yes 1, no 0	
	X8-7 Promotion of rural sports activities	Whether the policy addresses the objective of promoting physical activity in the countryside: yes 1, no 0	
	X8-8 Improvement of rural fitness facilities	Whether the policy addresses the objective of improving fitness facilities in the countryside: yes 1, no 0	
	X8-9 Building a sports powerhouse	Whether the policy addresses the goal of building a strong sporting nation: yes 1, no 0	
X9 Policy evaluation	X9-1 Full and accurate	Whether the policy is informative: yes 1, no 0	Revised based on research by Cheng Meichao, Cao Wenxiao, and others
	X9-2 Clear-cut	Whether the policy is well targeted: yes 1, no 0	
	X9-3 Clear-cut responsibilities and authority	Whether the policy has clear lines of responsibility and authority: yes 1, no 0	
	X9-4 Program feasibility	Whether the policy program is viable: yes 1, no 0	

### 3.3 Research methodology

#### 3.3.1 Concept of PMC index model

The PMC index model, first proposed by Mario Arturo Ruiz Estrada, argues that policy evaluation models cannot ignore any relevant variables (30). The model measures and evaluates the internal consistency of a single policy by constructing indicators of relevant variables. Then, the model reflects the differences between the policy as a whole and various aspects by analyzing the strengths and weaknesses of a single policy, thereby measuring the effectiveness and impact of the policy. In 2015, Prof. Zhang Yong’an introduced the PMC index model into China, and thus far, the model has become one of the most important research methods in the quantitative study of policy literature.

#### 3.3.2 PMC index model construction

The PMC index model consists of five basic steps: (1) variable classification and parameter identification; (2) establishment of a multi-input–output table; (3) PMC index calculation; (4) PMC surface diagram construction; (5) a comprehensive analysis of policy strengths and weaknesses, based on criteria (33).

1. The steps of the PMC index model calculation process are as follows:

① Construct variables according to the rural sports policy text, including primary variables and secondary variables: X ~ N (0, 1).

② Establish a multi-input–output table, and according to the rural sports policy text of the second-level variables, assign a specific value of 0 or 1. If the content of the policy involves the variable, the content is assigned the value of 1; otherwise, 0. This is as shown in Equation 1:


(1) $$X=\left\{XR:\left[0\sim 1\right]\right\}\text{.}$$

③ Calculate the value of the first-level variable, according to the second-level variable parameters, to take the value of the cumulative, as shown in Equation 2:


(2) $$X_{t}=\overset{n}{\underset{j=1}{\sum }}\frac{X_{tj}}{TX_{tj}}\;t=1,2,3,4,5,6,7,8,9,10$$

where t is a level 1 variable; j is a level 2 variable, T is the number of the tth level 1 indicator, and n is the number of level 2 evaluation variables under a particular level 1 evaluation variable. In this paper, the minimum value of n is 3, and the maximum value is 11.

④ To calculate the final PMC index for a given policy, the level 1 variables derived in the previous step are summed up and calculated as in Equation 3:


(3) $$\begin{array}{l} PMC=X1+X2+X3+\ldots +X10=\left(\overset{n}{\underset{j=1}{\sum }}\frac{X_{1}j}{T\left(X_{1}j\right)}\right)+\\ \;\left(\overset{n}{\underset{j=1}{\sum }}\frac{X_{2}j}{T\left(X_{2}j\right)}\right)+\left(\overset{n}{\underset{j=1}{\sum }}\frac{X_{3}j}{T\left(X_{3}j\right)}\right)+\cdots +\\ \;\left(\overset{n}{\underset{j=1}{\sum }}\frac{X_{9}j}{T\left(X_{9}j\right)}\right)+X_{10} \end{array}$$

where j is the secondary variable, and T is the number of secondary variables.

1. Criteria for classifying rural sports policies.

The calculation results of the PMC index can provide strong support for judging the consistency level of the focal policy. The total index score of each rural sports policy is 9 points, which is modified with reference to the policy evaluation criteria used in previous studies, such as Dong Jichang (34). In this study, based on the size of the policy PMC value, the quality grade of rural sports policies is divided into four grades: excellent, good, acceptable, and unqualified. The criteria for dividing the grades are shown in Table 2.

**Table 2:** PMC index scores and corresponding policy ratings.

PMC index score	Policy ratings
7 ~ 9	Excellent
5 ~ 6.99	Good
3 ~ 4.99	Acceptable
0 ~ 2.99	Unsatisfactory

#### 3.3.3 Construction of PMC surface diagram

The PMC surface diagram not only can show the quantitative assessment results of the PMC index in a three-dimensional and intuitive way but can also reflect the scores of different assessment dimensions of the policy according to different color areas. PMC surfaces are typically uneven three-dimensional forms. The convex portion of the surface implies a higher score for the corresponding evaluation indicator of the policy, whereas the concave part indicates a lower score for the corresponding evaluation indicator (35). In this paper, the PMC surface diagram is transformed into a 3*3 matrix from the specific values of nine level 1 variables. The diagram is drawn by MATLAB, and the calculation is shown in Equation 4.


(4) $$PMC\left(\mathrm{meshes}\right)=\left[\begin{array}{ccc} P1 & P2 & P3\\ P4 & P5 & P6\\ P7 & P8 & P9 \end{array}\right]$$

## 4 Results and discussion

### 4.1 Research results

#### 4.1.1 Policy screening results

The PMC index model can comprehensively analyze and evaluate any one policy, on the basis of ensuring (as much as possible) the plurality and comprehensiveness of the sample policies in terms of the form of issuance, policy theme, time of issuance, issuing agency, etc. Therefore, this study selects 20 representative policies as the evaluation object from the 56 policies collected above (see Table 3). These include six at the national level (p1–p6), six at the provincial level (p7–p12), and eight at the city and county level (p13–p20) as the final samples to be analyzed.

**Table 3:** Chinese rural sports policy texts.

Serial number	Name of policy	Publication date	Issuing body
P1	Provisional regulations for rural sports work	2002	State General Administration of Sport, Ministry of Agriculture
P2	Circular on the issuance of the opinions on the implementation of the Farmers’ Sports and Fitness Project	2006	State General Administration of Sport
P3	Circular on the issuance of the opinions on further strengthening rural sports work by functioning as township comprehensive cultural stations	2010	State General Administration of Sports, Ministry of Culture, Ministry of Agriculture
P4	Guidelines on further strengthening of farmers’ sports	2017	Ministry of Agriculture, State General Administration of Sport
P5	Guiding opinions on promoting the high-quality development of farmers’ sports in the 14th Five-Year Plan	2022	Ministry of Agriculture and Rural Affairs, General Administration of Sport, National Rural Revitalization Authority
P6	Guidelines on promoting the work of sports for rural revitalization	2023	State General Administration of Sports, Central Civilization Office, National Development and Reform Commission, Ministry of Education, National People’s Committee, Ministry of Finance, Ministry of Housing and Urban–Rural Development, Ministry of Agriculture and Rural Development, Ministry of Culture and Tourism, National Health and Wellness Commission, Central Committee of the Communist Youth League, All-China Women’s Federation
P7	Measures for the implementation of the Shandong Province farmers’ sports and fitness project	2006	Shandong Provincial Sports Bureau
P8	Implementation program of the 2013 Jiangxi Provincial farmers’ sports and fitness project	2013	Jiangxi Sports Bureau
P9	Notice on strengthening the implementation of the guidelines for the maintenance and management of rural public sports facilities in Shanxi Province	2014	Shanxi Provincial Sports Bureau
P10	Chongqing Municipal Sports Bureau on the construction of farmers’ sports and fitness project in 2016	2016	Chongqing Sports Bureau
P11	Zhejiang Province sports into rural cultural hall three-year action plan (2020–2022) notice,	2020	Zhejiang Provincial Sports Bureau
P12	guidelines of Shanghai Municipal Sports Bureau on further promoting the construction of rural sports and fitness facilities in the city	2021	Shanghai Agriculture and Rural Committee
P13	Circular on the working opinions of Fuzhou Municipality on the activities of the “Year of Rural Sports.”	2004	General Office of Fuzhou Municipal People’s Government
P14	Notice on the issuance of the implementation of opinions on promoting the “Hundred Villages Project” for rural sports facilities	2006	Chengde City Government, Hebei Province
P15	Jingjiang City, Taizhou City, Jiangsu Province on strengthening the implementation of rural sports work in Jingjiang City in 2013	2013	Office of the People’s Government of Jingjiang City
P16	Notice of the implementation program for the construction of a 20-min sports and fitness circle in rural Taizhou City, Jiangsu Province	2014	Taizhou Municipal People’s Government Office
P17	Notice on the main points of farmers’ sports work in Longhai City, Zhangzhou City, Fujian Province in 2016	2016	Office of the People’s Government of Longhai City
P18	Implementation Program of the farmers’ sports and fitness project at rural and village levels in Guizhou Qiannan Prefecture (2018–2020)	2018	Office of the People’s Government of Qiannan Prefecture
P19	Notice on the issuance of Haiyan County sports in rural cultural halls three-year action plan (2020–2022)	2020	CPC Haiyan County Committee Propaganda Department Haiyan County Culture and Radio, Television, Tourism, and Sports Bureau
P20	Notice on the issuance of the Zhoushan City sports into fisheries and rural cultural auditorium 2022 implementation Program	2022	Zhoushan Municipal Bureau of Culture, Radio, Television, Tourism, and Sports

#### 4.1.2 Calculation results of the PMC index

In this paper, the data from 20 samples of rural sports policies were integrated into a multiple input–output table through text mining (see Table 4). All the secondary variables have the same weights, and if the policy text meets the requirements of the evaluation index of the secondary variables, that text will take the value of “1”; otherwise, “0.” The PMC index of each rural sports policy was calculated according to the multi-input–output table, and the PMC index score of each policy was obtained (see Table 5). According to the quantitative evaluation results of the PMC index model, the specific value of the PMC index of each policy and its ranking and grade can be obtained. These values, rankings, and grades, in turn, reveal the overall quality level of the sample policies.

**Table 4:** Multi-input–output table for rural sports policy.

Level 1 variable	Level 2 variable	P1	P2	P3	P4	……	P18	P19	P20
X1	X1.1	0	0	0	1	……	1	1	1
	X1.2	1	1	1	1	……	1	1	1
	X1.3	1	1	1	1	……	1	1	1
	X1.4	1	1	0	1	……	1	1	1
	X1.5	1	1	1	1	……	0	1	1
X2	X2.1	1	1	1	1	……	0	0	0
	X2.2	0	0	0	0	……	0	0	0
	X2.3	0	0	0	0	……	1	1	1
X3	X3.1	1	1	0	0	……	1	1	1
	X3.2	1	1	1	1	……	1	1	1
	X3.3	1	1	1	1	……	1	1	1
	X3.4	1	1	0	1	……	1	1	1
X4	X4.1	0	0	0	O	……	0	1	1
	X4.2	0	1	0	0	……	1	0	0
	X4.3	1	0	1	1	……	0	0	0
X5	X5.1	1	1	1	1	……	1	1	1
	X5.2	1	0	1	1	……	0	1	1
	X5.3	0	0	1	1	……	0	1	1
	X5.4	1	1	1	1	……	1	1	1
	X5.5	1	1	1	1	……	0	1	1
	X5.6	0	0	1	1	……	1	1	1
	X5.7	0	1	1	1	……	0	1	1
	X5.8	0	0	0	1	……	0	0	1
	X5.9	1	1	1	1	……	0	1	1
X6	X6.1	1	1	1	1	……	1	1	1
	X6.2	0	1	1	1	……	1	1	1
	X6.3	1	1	1	1	……	1	1	1
	X6.4	0	1	1	1	……	1	1	1
	X6.5	1	0	0	1	……	1	1	1
X7	X7.1	1	1	1	1	……	1	1	1
	X7.2	1	1	1	1	……	1	1	1
	X7.3	0	1	1	1	……	0	1	1
	X7.4	1	0	1	1	……	1	1	1
X8	X8.1	1	1	1	0	……	1	1	1
	X8.2	0	0	0	1	……	0	1	1
	X8.3	0	1	1	1	……	1	1	1
	X8.4	0	0	0	0	……	0	1	0
	X8.5	0	0	0	0	……	1	1	0
	X8.6	0	1	1	1	……	0	1	1
	X8.7	1	0	1	1	……	0	1	1
	X8.8	1	1	1	1	……	1	1	1
	X8.9	0	0	0	0	……	0	1	0
X9	X9.1	0	1	1	1	……	1	1	1
	X9.2	0	0	1	1	……	1	1	1
	X9.3	1	1	1	1	……	1	1	1
	X9.4	1	1	1	1	……	1	1	1

**Table 5:** Quantitative evaluation results of PMC index model for rural sports policies in China.

	X1	X2	X3	X4	X5	X6	X7	X8	X9	PMC index per policy	Per policy depression index	Hierarchy	Rankings
P1	0.80	0.33	1.00	0.33	0.56	0.60	0.75	0.33	0.50	5.21	3.79	Good	15
P2	0.80	0.33	1.00	0.33	0.56	0.80	0.75	0.44	0.75	5.77	3.23	Good	13
P3	0.60	0.33	0.50	0.33	0.89	0.80	1.00	0.56	1.00	6.01	2.99	Good	11
P4	1.00	0.33	0.75	0.33	1.00	1.00	1.00	0.56	1.00	6.97	2.03	Good	7
P5	1.00	0.33	1.00	0.33	1.00	1.00	0.75	1.00	1.00	7.42	1.58	Excellent	4
P6	1.00	0.33	1.00	0.33	1.00	1.00	1.00	1.00	1.00	7.67	1.33	Excellent	1
P7	0.80	0.33	1.00	0.33	1.00	1.00	1.00	0.67	1.00	7.13	1.87	Excellent	6
P8	0.60	0.33	0.75	0.33	0.67	0.80	0.75	0.44	1.00	5.68	3.32	Good	14
P9	0.40	0.33	0.25	0.33	0.33	0.60	0.50	0.22	0.25	3.22	5.78	Acceptable	19
P10	0.20	0.33	0.25	0.33	0.22	0.40	0.50	0.33	0.50	3.07	5.93	Acceptable	20
P11	1.00	0.33	1.00	0.33	0.89	1.00	1.00	0.89	1.00	7.44	1.56	Excellent	3
P12	0.60	0.33	0.50	0.33	0.44	0.80	0.75	0.67	0.75	5.18	3.82	Good	16
P13	0.60	0.33	1.00	0.33	1.00	0.80	0.75	0.67	0.75	6.23	2.77	Good	10
P14	0.40	0.33	0.50	0.33	0.22	0.40	0.50		0.75	3.88	5.12	Acceptable	18
P15	1.00	0.33	1.00	0.33	0.78	0.80	0.50	0.56	1.00	6.30	2.70	Good	9
P16	0.80	0.33	1.00	0.33	0.67	1.00	0.75	0.56	1.00	6.44	2.56	Good	8
P17	0.40	0.33	0.75	0.33	0.56	0.60	0.50	0.44	0.75	4.67	4.33	Acceptable	17
P18	0.80	0.33	1.00	0.33	0.33	1.00	0.75	0.44	1.00	5.99	3.01	Good	12
P19	1.00	0.33	1.00	0.33	0.89	1.00	1.00	1.00	1.00	7.56	1.44	Excellent	2
P20	1.00	0.33	1.00	0.33	1.00	1.00	1.00	0.67	1.00	7.33	1.67	Excellent	5

As shown in Table 5, it can be seen that the 20 representative policies are ranked from highest to lowest based on the PMC-Index: P6 > P19 > P11 > P5 > P20 > P7 > P4 > P16 > P15 > P13 > P3 > P18 > P2 > P8 > P1 > P12 > P17 > P14 > P9 > P10. Among them, there are 4 policies of excellent grade, 10 of good grade and 4 of acceptable grade. With 70 percent of them being excellent and good, and no unqualified policies existing, it indicates that the quality of rural sports policies in China is generally high.

Table 6 shows that the overall average PMC index score of the 20 rural sports policies selected in this paper is 5.96; the national policy score is 6.51, the provincial level is 5.29, and the urban and county level is 6.05. Among them, there are four policies with an excellent rank, 10 policies with a good rank, and four policies with acceptable rank, and the ratio of excellent and good is 70%, and there is no unqualified policy. These findings show that the quality of China’s rural sports policies is higher in general. From the perspective of evaluation indicators, the mean value of the primary indicators in the selected sample of 20 policies is 0.66, and only the policy grade (X2), policy timeliness (X4), and policy objective (X8) are below the average value. Since all the secondary variables have the same weight value, if the policy text meets the requirements of the evaluation index of the secondary variables, it will take the value of “1”; otherwise, “0.” The secondary variables of the policy level (X2) are national, provincial, municipal, and county level, and the secondary variables of the policy timeliness (X4) are long-term, medium-term, and short-term, all of which are options that can only be selected as 1Therefore, the PMC scores of X2 and X4 can only be 0.33, showing a large defect. The mean value of the policy objective (X8) is 0.59, which is lower than the average value. Upon analyzing policy documents, it is evident that the primary focus of sample policies is to enhance public sports facilities and promote the development of rural sports activities. However, there is a lack of emphasis on national strategic objectives such as rural revitalization and the advancement of sports prowess. This shows that the current objective setting in the process of rural sports policy formulation is not perfect and needs to be strengthened.

**Table 6:** Quantitative mean evaluation results of PMC index model of rural sports policy in China.

	X1	X2	X3	X4	X5	X6	X7	X8	X9	PMC index per policy	Per policy depression index	Hierarchy
Country-level averages	0.87	0.33	0.88	0.33	0.83	0.87	0.88	0.65	0.88	6.51	2.49	Good
Provincial averages	0.60	0.33	0.63	0.33	0.59	0.77	0.75	0.54	0.75	5.29	3.60	Good
Urban county level averages	0.75	0.33	0.91	0.33	0.68	0.83	0.72	0.62	0.91	6.05	2.95	Good
Overall average	0.74	0.33	0.81	0.33	0.70	0.82	0.78	0.59	0.85	5.96	3.04	Good

**Figure 3:**
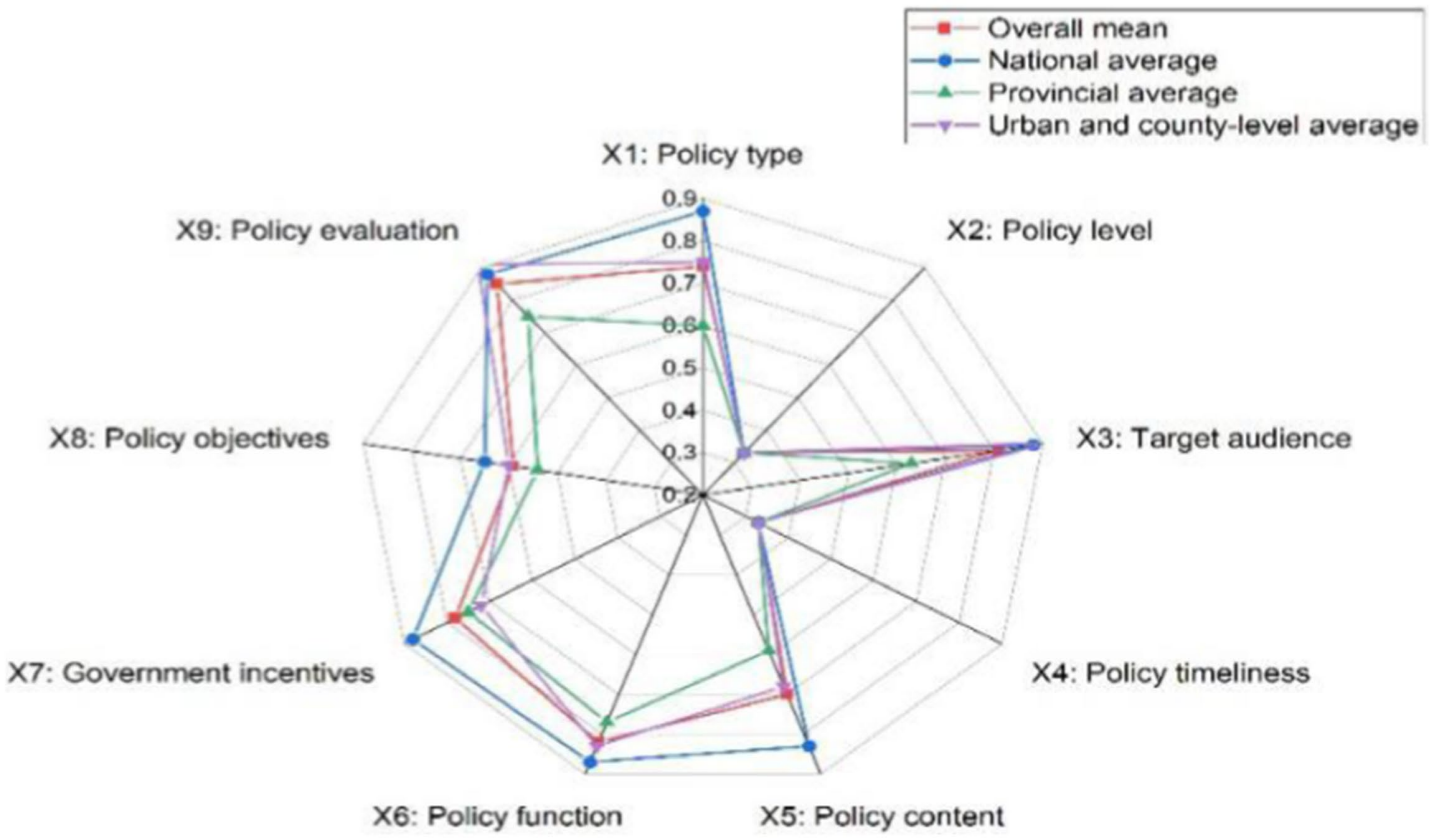
Dybla diagrams of the means of the three levels of the sample of rural sports policies in China.

In addition, in order to more intuitively show the overall scores of rural sports policies, this paper compares the average scores of rural sports policies at three levels. Dybla diagrams are then used to display those scores. From Figure 3, a large gap can be seen in the PMC scores of rural sports policies; the overall mean of rural sports policies at the national level is the highest, followed by the city and county levels, while the provincial level is the smallest. The state pays particular attention and focus to rural sports and is able to issue a series of instructions in a comprehensive and balanced manner. City and county areas are also better able to implement the policies. Conversely, provincial policies have collapsed, with all level 1 indicators below the average. This finding clearly shows that the consistency and effectiveness of rural sports policy implementation needs to be improved.

#### 4.1.3 Quantitative evaluation analysis of rural sports policies at different levels

##### 4.1.3.1 Empirical analysis of national-level rural sports policies

Table 5 shows that P1-P6 are national-level rural sports policies, with PMC indexes of 5.21, 5.77, 6.01, 6.97, 7.42, and 7.61. Of these, four policies perform well, and two are excellent, with a mean value of 6.51 and a concave index of 2.49. Figure 3 shows that all the level 1 indexes of the national-level policies, except for the policy level and the timeliness of the policies, are higher than the average value. Through collating the policy texts, the national-level rural sports policies are found to not only include rural sports facilities, sports activities, fitness guidance, sports personnel training, policy and institutional safeguards; they, also put forward clear policy objectives and give detailed and feasible program guidance, which shows that the overall design of the national-level rural sports policies is more reasonable.

**Figure 4:**
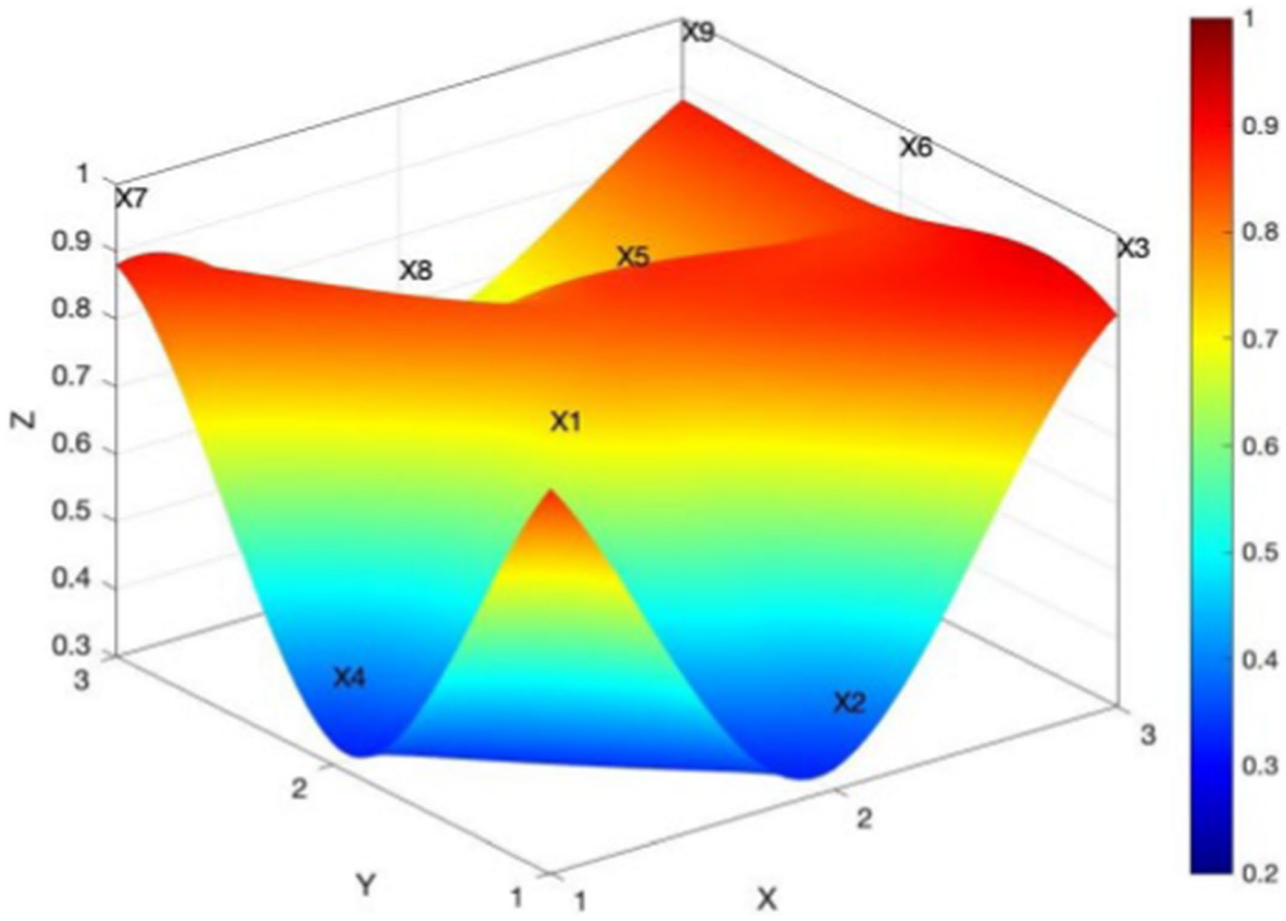
PMC surface of the average value of policies at the national level.

**Figure 5:**
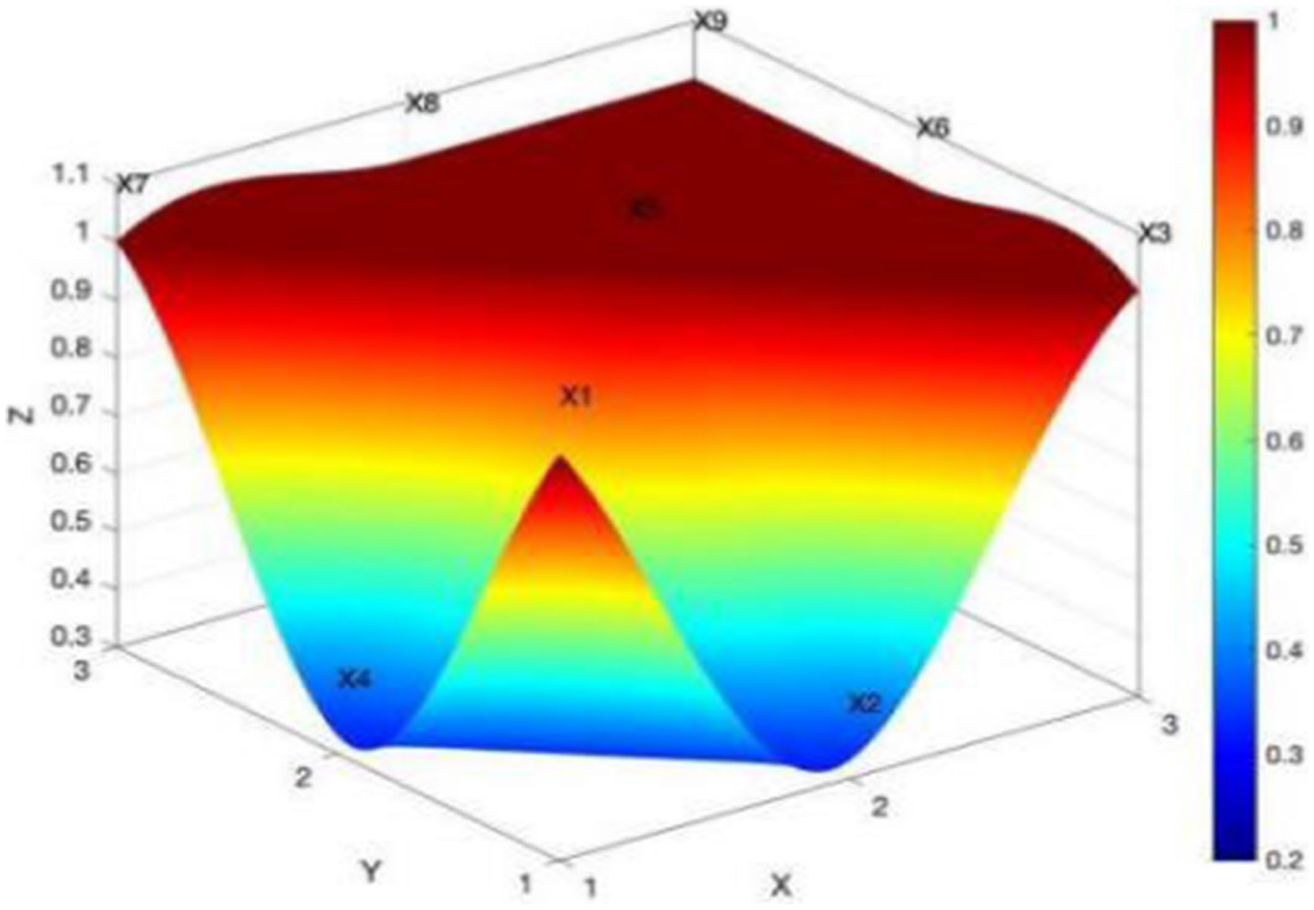
PMC surface of the highest scoring policy P6.

The PMC surface diagram can show the scores of each studied policy on each variable from a three-dimensional perspective. This helps to analyze the advantages and shortcomings of China’s rural sports policies. The different colored blocks in the PMC surface diagram also represent the scores of different indexes corresponding to the variables. The higher the surface is in the three-dimensional coordinates, the smaller is the degree of concavity of the surface, and the more obvious is the black part of the surface, the more comprehensive the indexes involved in the policy are, and the higher the evaluation grade of the policy is (36). As seen in Figure 4, the PMC surface of the national rural sports policy is very smooth, the degree of concavity is low, and the surface is at a higher level of the three-dimensional coordinates. This shows that the internal consistency of the national rural sports policy is high, and the structure is reasonable. Figure 5 shows Policy P6, which is a national-level policy, with the highest PMC index score. The black part of its surface is very obvious, showing that the indicators involved in P6 are more comprehensive, and the policy implementation is effective.

##### 4.1.3.2 Empirical analysis of provincial rural sports policies

**Figure 6:**
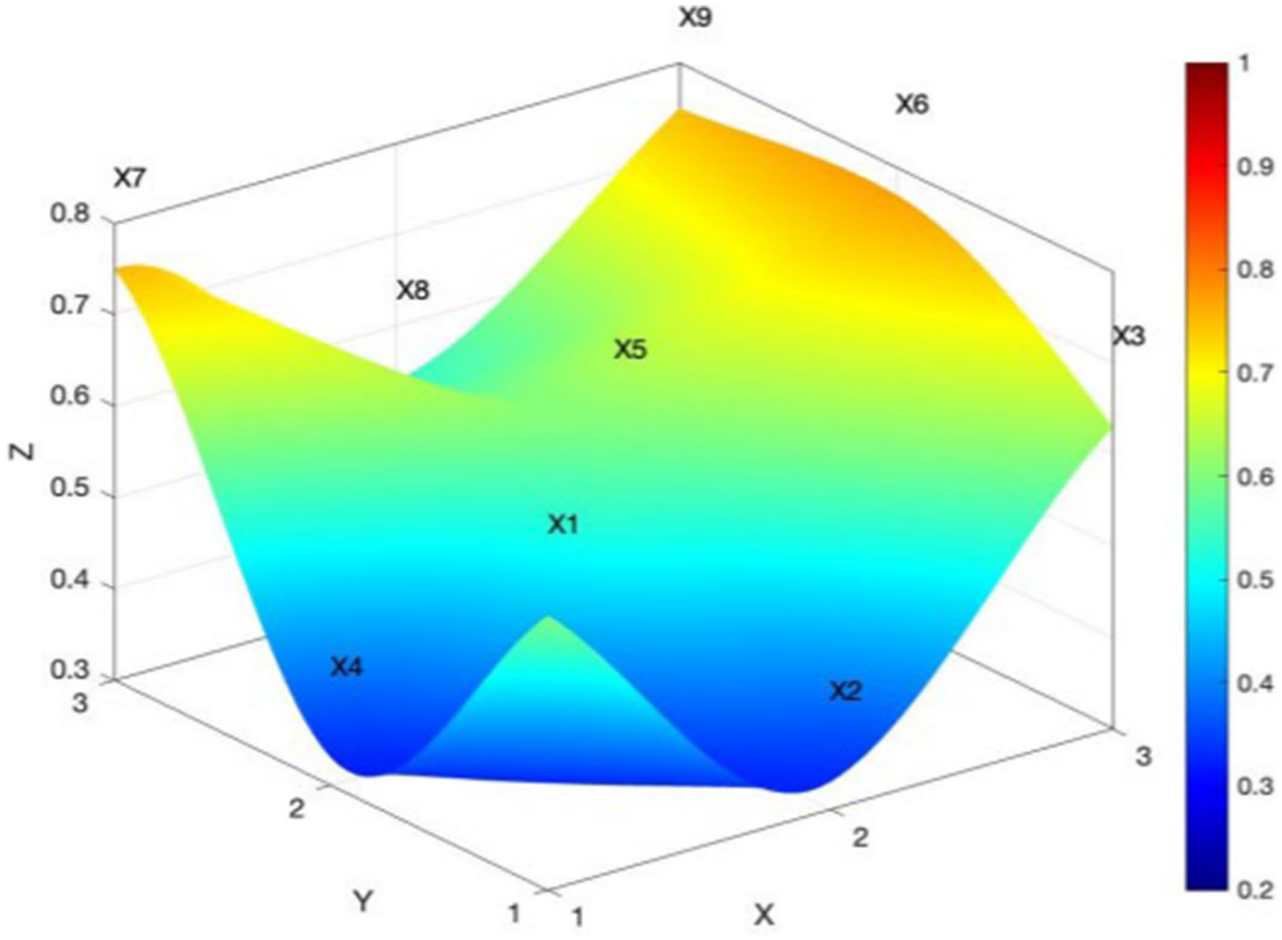
PMC surface of the average provincial rural sports policies.

Policies P7-P12 are provincial rural sports policies, with PMC indexes of 7.13, 5.68, 3.22, 3.07, 7.44, and 5.18, respectively. Two of the six policies are excellent, two are good, and two are acceptable, with an overall provincial policy mean of 5.29 and a concavity index of 3.6. This means that the internal consistency of the policies is low. From Figure 6, one can see that all the variables of provincial rural sports policies are more seriously concave in the surface diagram. Also, all the first-level indicators are lower than the average value, especially in the type of policy, policy content, and policy objectives, which have the greatest degree of concavity. Based on the multi-input–output table and policy content text analysis, provincial rural sports policies mostly relate to farmers’ sports, public fitness facilities, national fitness, fitness guidance, etc. However, these policies lack sports personnel training, building public services for national fitness, and other features. In terms of policy objectives, the policies are mainly about perfecting public sports facilities and promoting rural sports activities; they seldom involve national strategic objectives, such as rural revitalization and a strong sporting country. In terms of policy guarantees, the above policies are mostly about improving public sports facilities and promoting rural sports. Again, they rarely involve rural revitalization and a strong sporting country. The policy guarantees are mostly about government services and the provision of special financial support, but there is a lack of certain legal and regulatory guarantees.

**Figure 7:**
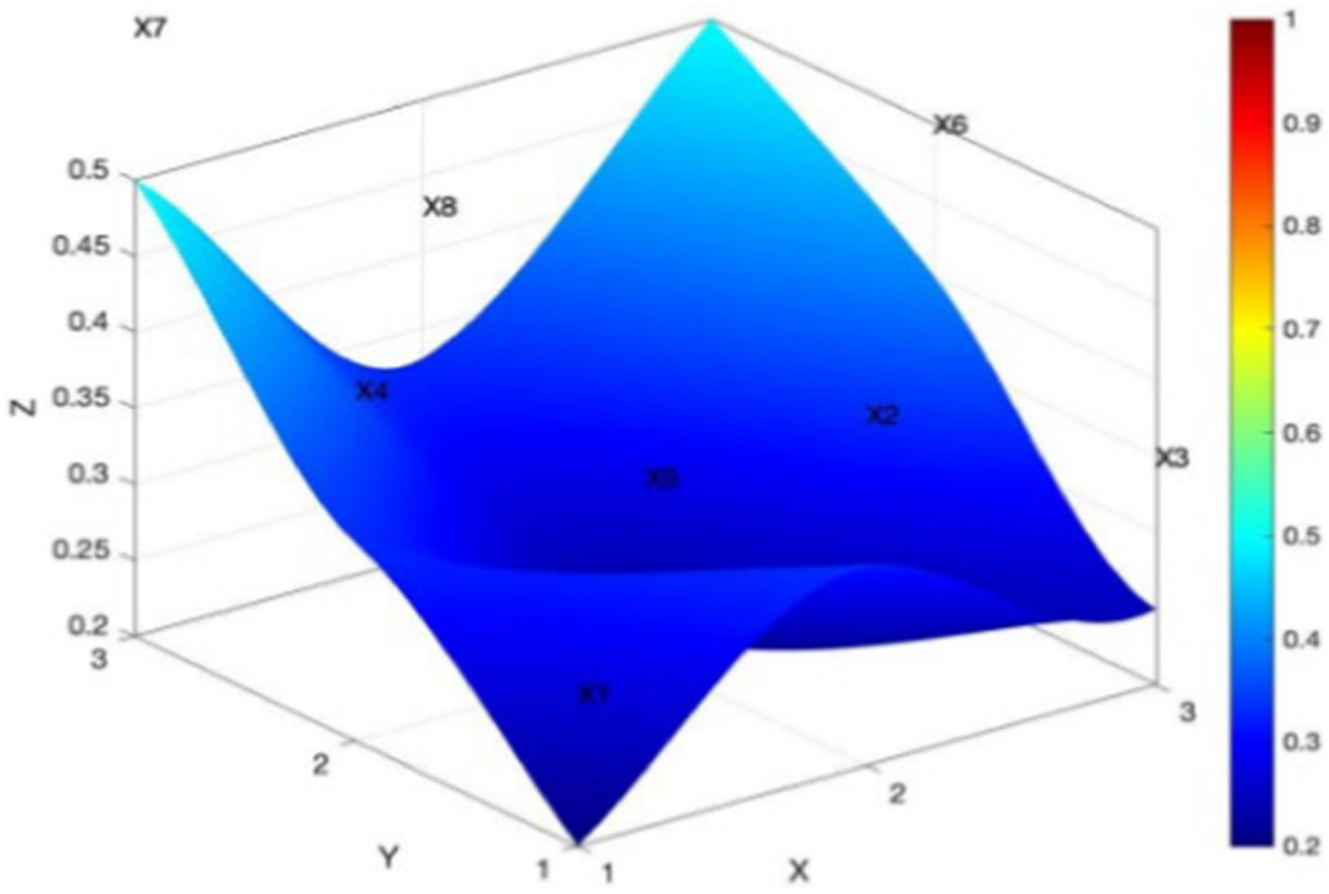
PMC surface diagram for minimum policy P10.

In Figure 7, P10 is the policy with the lowest PMC index score among all the policies, with a serious concave surface and at a lower level of the three-dimensional coordinates. Text combing revealed that P10, for the Chongqing Municipal Sports Bureau, issued the “Chongqing Municipal Sports Bureau on the construction of farmers’ sports and fitness project in 2016 notice.” The policy’s content only mentioned public sports facilities and rural sports activities. However, the guidance of fitness activities, sports personnel training, the corresponding system to ensure involvement, and normative guidance and institutional constraints on the function of the policy were all lacking. The policy’s evaluation of the responsibilities and power structure is not clear, and in short, the content is not detailed enough. In terms of policy functions, there is a lack of normative guidance and institutional constraints, and in terms of policy evaluation, responsibilities for implementing the policy and the policy’s powers are neither clear nor detailed.

##### 4.1.3.3 Empirical analysis of rural sports policies at urban county level

**Figure 8:**
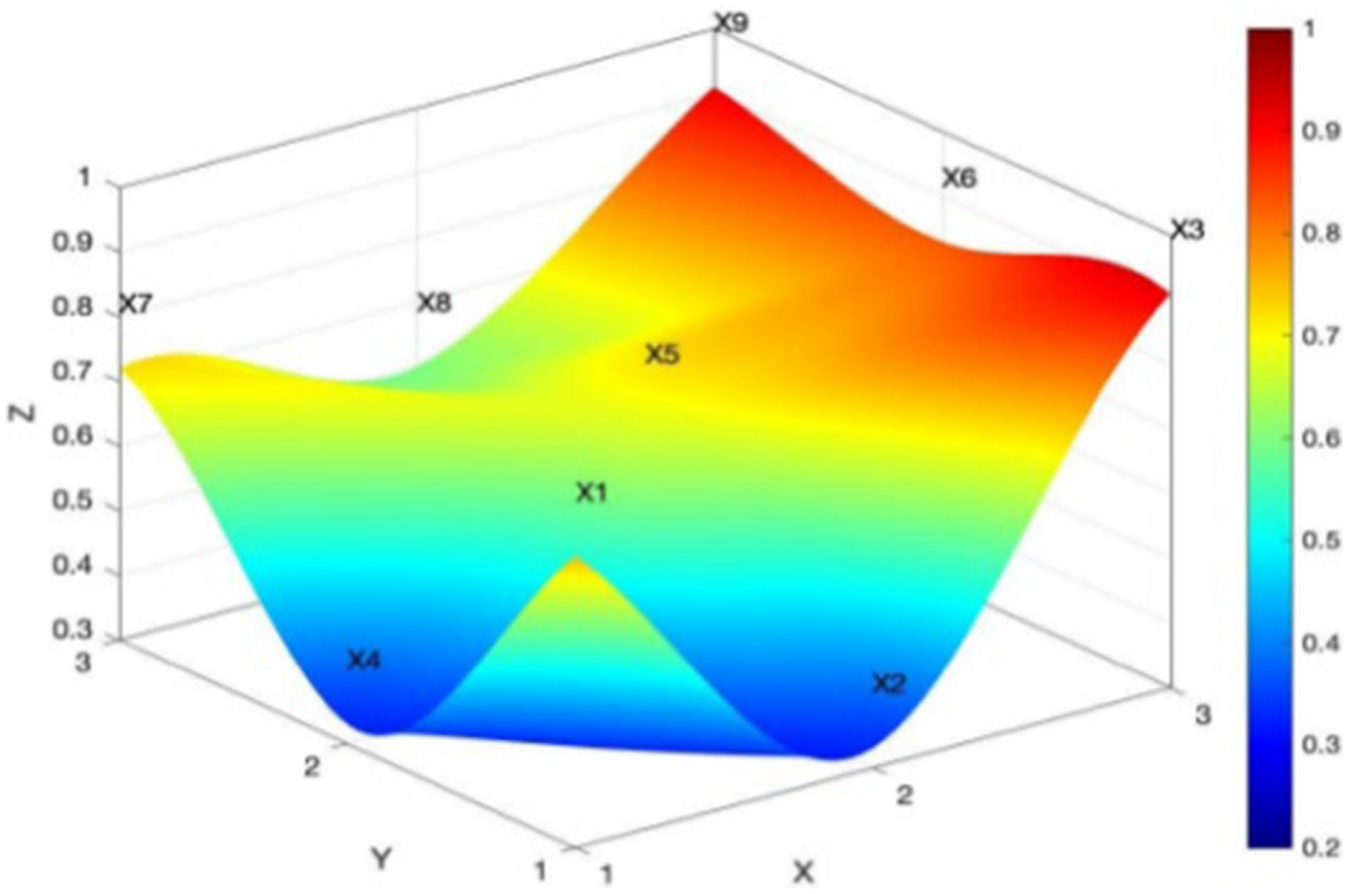
PMC surface of mean rural sport policy at city and county level.

Policies P13-P20 are rural sports policies at the urban county level, with a mean PMC index of 6.05 and a concave index of 2.95. The PMC index scores of each policy are 6.23, 3.88, 6.30, 6.44, 4.67, 5.99, 7.56, and 7.33, respectively. Of the eight policies, two performed well, four good, and two were acceptable. The overall performance of those policies at urban county level was good. From Figure 8, one can see that the PMC surface of rural sports policies at the urban and county levels is smoother, with a lower degree of concavity. This indicates that the rural sports policies at the urban and county levels have high internal consistency and a reasonable structure and that these places can better formulate and implement rural sports policies.

Overall, China’s rural sports policies are better designed and implemented at the national and urban county levels, but there are serious collapses at the provincial level. The collapse of provincial policies has led to a low overall score for rural sports policies. This finding suggests that there are fault lines and inconsistencies in the design and implementation of China’s rural sports policies, which in turn have led to ineffective policy implementation. It was disclosed through the interviews that the reason for this is that the majority of provinces merely “pass on the message” when implementing policies, without formulating corresponding policies in accordance with the spirit of national policies and the actual conditions of their own provinces. Some affluent provinces pay more attention to the development of urban sports, while neglecting the needs of sports in rural areas. In addition, the mechanisms for managing rural sports at the provincial level may be relatively weak, with problems such as inadequate supervision and assessment. This leads to a lack of effective communication and coordination in the process of policy implementation, which in turn makes it difficult for policies to be implemented in a comprehensive, timely, and effective manner. Therefore, even if the policy design process is idealized, the policy cannot completely avoid the deviations arising from the dynamic process of implementation. Attention to coherence and consistency in the process of policy implementation will enable policy implementation and execution to continuously adapt in a complex and changing environment. In order to promote the efficient realization of policy goals, on the one hand, policy sequencing can be achieved by putting theoretically superior but more acceptable policies ahead of ambitious policies (37). On the other hand, enhancing the acceptance of those for whom the policy is implemented is also an effective path.

### 4.2 Discussion

By further summarizing the results of the PMC index analysis of 20 representative sports policies in rural China, we can discuss the findings of this study.Among the 20 representative policy texts, excellent, good, acceptable and failing are 4, 10, 4 and 0 respectively. This is consistent with previous findings in the literature and suggests that the overall design of rural sports policies in China is relatively sound. However, there is a need to improve the coherence and effectiveness of rural sports policy implementation in China in terms of policy design and execution. The national level demonstrates the best performance, followed by urban and rural areas, with provincial levels showing the least effective implementation. This finding deviates from existing literature on this topic. For example, Du Hohua (38) on Chinese government’s open data policy and Wenjing and Ruilin (25) in her study of China’s ice and snow industry policy both concluded that municipal level > provincial level > national level. In contrast, Chongyan et al. (28), in her study of youth physical health promotion policies came to a conclusion that contradicts those two scholars: i.e., national level > provincial level > prefecture and city levels.

Since the formulation and implementation of each policy is limited and influenced by various factors, these influences work together to shape the outcome, implementation, and effectiveness of the policy (39). Therefore, this study investigated the implementation of rural sport policies by conducting interviews with two staff members from the Policy Department of the State General Administration of Sport, the China Farmers’ Sports Association, the Hainan Sports Bureau, the Guangxi Sports Bureau, the Rural Sports and Cultural Center of Wenli Township, Lingshan County, Qinzhou City, Guangxi, and the Sports and Cultural Station of Qiannan Prefecture, Guizhou (see Appendix 1 for an outline of the interviews). The interviews were carried out with staff from national associations as well as provincial, prefecture and county sports management organizations. This approach contributed to a top-down understanding of how rural sports policies are implemented in China and also shed light on any obstacles that may hinder their successful implementation. The interviewees indicated that current rural sports policies have achieved remarkable results in their implementation process. For example: Rural sports facilities have been greatly improved to provide better conditions for residents to participate. The cohesion and execution of grassroots sports organizations have been enhanced to provide strong protection for rural sports development. The level of participation by rural residents has significantly increased creating a strong sports atmosphere. However, numerous difficulties and challenges still persist during the implementation of policies. Firstly, there are resource allocation issues leading to constraints such as inadequate funding and venues that cannot meet the needs of all residents. Secondly, biases in understanding certain regions have hindered policy implementation. Thirdly, there is a lack of clear division of responsibilities and labor among national, provincial, and county governments in implementing rural sports policies, resulting in insufficient supervision and evaluation. Additionally, the majority of provincial-level institutions prioritize performance over providing real services, which leads to a problematic direction for policy implementation. To improve the effectiveness of China’s rural sports policy implementation, this study proposes several suggestions: first, enhance the understanding and attention given by national and local governments to rural sports policies to ensure they are formulated according to actual rural conditions; secondly, rationalize resource allocation to ensure that funds and venues can adequately meet the sporting needs of rural residents; thirdly, strengthen policy publicity and education efforts to enhance rural residents’ understanding and acceptance of sports policies; fourthly, focus on real service delivery rather than just performance at provincial-level organizations; fifthly, strengthen policy publicity and education to improve rural residents’ understanding and recognition of sports policies; finally, establish a sound monitoring and evaluation mechanism to facilitate effective communication and coordination in the process of policy implementation, and truly realize the original intention of the policy to serve people’s livelihoods.

Compared with existing literature, the contribution of this paper is mainly reflected in two aspects. The first is the research methodology. Existing research on China’s rural sports policy is relatively rich, but the research methods used have been relatively traditional and single, mostly focusing on qualitative exploration. This study adopts the PMC index model, which advocates that all relevant variables should be included as far as possible and that all variables are equally important. In addition, the variables should be regarded as dichotomous variables, in order to overcome the shortcomings of existing policy evaluation methods that pay too much attention to some variables and ignore others. The second contribution is the research perspective. Existing studies mostly focus on the changing characteristics and influencing factors of rural sports policies, but few studies focus on the design and implementation of rural sports policies. Although China’s rural sports policies have effectively promoted the development of rural sports, problems still exist, such as differences in administrative hierarchy and regions. The consistency and effectiveness of policy implementation also need to be improved. Therefore, it is urgently important to study the design and implementation of rural sports policies in China. This thesis analyzes the two dimensions of policy design and implementation, which is conducive to improving the rural sports policy system and promoting the high-quality development of rural sports.

Although the PMC index can reasonably quantify the attributes of the dimensions of rural sports policies, research on the design and implementation of rural sports policies still has some limitations. Firstly, the PMC index model cannot properly evaluate the implementation effects of each policy. Secondly, this study is mainly a textual analysis of existing policies; there is less focus on the relationship between policy formulation and implementation. Thirdly, farmers play a crucial role in determining their own physical well-being and are therefore the focal point of rural sports policy formulation. It is imperative that the policy prioritizes meeting the needs of farmers and aims to promote their comprehensive development. The ultimate goal of any farmer sports policy should be to encourage greater participation from farmers in shaping its effectiveness, although further research into this area is still required.

## 5 Conclusion

This study conducts an exploratory quantitative evaluation of rural sports policies introduced in China, based on the PMC model. Firstly, 56 policy documents included in the study were text-mined; ROST CM6 software was used to statistically analyze the social network mapping of high-frequency words. Then, the PMC index model for rural sports policy evaluation was constructed, based on the text mining results. Ultimately, 20 representative policies were evaluated for quality. The study conclusions are as follows: (1) the overall design of China’s rural sports policies is relatively reasonable. Among the 20 representative policy texts, 4 are deemed excellent, 10 are considered good, and 4 are acceptable. There are no unqualified policies, indicating that China’s rural sports policies demonstrate a certain degree of scientific rigor and effectiveness. (2) The overall sample of 20 policies selected for this study is considered to be of a high standard. Among them, the mean values of audience target, policy function and policy evaluation reached an excellent level, indicating that China’s rural sports policies demonstrate a high degree of consistency in these areas. However, lower scores were found for policy type, policy content, policy guarantee and policy objective. This is generally consistent with the findings of Xiong et al. (32) study on China’s fire safety education policy and Fu Weizhong’s (40) study on construction waste resource recycling industry policy. (3) The implementation of rural sports policy in China needs to be improved in terms of consistency and effectiveness. The study indicates that the most effective design and implementation of China’s rural sports policies occur at the national level, followed by the urban and county levels, with the least effective being at the provincial level. This is attributed to the fact that China’s rural sports policy has a relatively comprehensive top-level design. National level policies provide overall and strategic guidance, and as they are implemented at lower administrative levels, rural sports policies become significantly more operational and comprehensive. However, management mechanisms at the provincial level are relatively weak, with insufficient monitoring and evaluation. This has resulted in a lack of effective communication and coordination in the implementation process at the provincial level, making it difficult to implement policies comprehensively, timely, and effectively.

In view of the above conclusions and combined with the development path of rural sports, this paper puts forward the following policy improvement suggestions: Firstly, Construct a multi-dimensional rural sports policy system: We can optimize the rural sports policy from the perspectives of enhancing the policy content, reinforcing the incentive mechanism, optimizing the policy function and attaining the policy goal setting. This can contribute to the construction of a sound and complete rural sports policy system and promote the high-quality development of rural sports in China. Meanwhile, Enhance the consistency and effectiveness of the implementation of the rural sports policy system: (1) establish national, provincial, urban, and county-level rural sports policy coordination agencies. Improve information sharing and communication mechanisms, and ensure that all levels of government understand and convey superior policy guidance in a timely manner. Efforts should also be made to respond to policy implementation in a timely manner and eliminate information asymmetry. (2) Clarify the responsibilities and division of tasks of national, provincial, and county-level governments in the implementation of rural sports policies. Ensure that all levels of government fulfill their responsibilities, and form a mechanism for work coordination and collaborative promotion. (3) Regularly convene joint meetings to examine the implementation of rural sports policies. Solve the problems and difficulties in the implementation process in a timely manner, and implement specific implementation plans for policies. (4) Establish a sound supervision and assessment mechanism. Specifically, evaluate and assess the implementation of rural sports policies by governments at all levels, identify problems in a timely manner, and take effective measures to correct deviations. These steps would improve and perfect policies. Based on evaluation results, encourage governments and relevant departments at all levels to actively promote the implementation of rural sports policies through incentive measures, such as rewards and commendations, thereby forming a positive cycle.
