# Supplementary material for: Policy design and implementation: a quantitative evaluation and empirical study of China’s rural sports policy based on the PMC index model
Source: Front Public Health. 2024 Aug 29;12:1430173. doi: 10.3389/fpubh.2024.1430173 (PMC11406341; doi:10.3389/fpubh.2024.1430173)
Supplement: Supplementary file 1 [file Data_Sheet_1.pdf]

Interview Outline

1. Introduction

1.1 Self-introduction and statement of purpose of the interview

1.2 The interviewee's essential details, including their name, job title, and area of responsibility.

2. Thematic discussion

2.1 Staff of the Policy Department of the General Administration of Sport of China

（1）Could you tell us about the main ideas and features of rural sports policy design in recent years?

（2）Please provide an overview of the background and objectives of the current rural sports policy.

（3）What are the main objectives and specific targets of the rural sports policy? How were these objectives and targets set?

（4）What are the main factors considered in the design of rural sports policies? For example, rural sports facility base, farmers' sports needs, local economic development, etc.

（5）In the process of policy design, how can the needs of rural sports in different areas and at different levels of development be balanced?

2.2 Staff of provincial/autonomous region/municipality sports bureaux

（1）In your province/autonomous region/municipality, which departments or organisations are responsible for the implementation of rural sports policy? What is the division of responsibilities among them?

（2）What is the current status of implementing the rural sports policy in your province/autonomous region/municipality?

（3）In the process of policy implementation, are there appropriate monitoring and evaluation mechanisms to ensure the effective implementation of the policy?

（4）What are the main challenges you have faced in implementing rural sports policy? How did you deal with them?

（5）How do you think to strengthen the cooperation and coordination between the

government, social organizations, farmers and other parties in the development of rural sports?

2.3 China Farmers' Sports Association, Inter-country Sports and Cultural Centre, Wenli Township, Lingshan County, Qinzhou City, Guangxi, Staff of Sports and Cultural Station, Qiannan Prefecture, Guizhou

（1）How is the development of rural sports and cultural activities in your area? What are the success stories?

（2） What is the situation of rural sports facilities in your area? Are there any problems such as insufficient investment and outdated equipment?

（3）What do you think needs to be improved in the current rural sports policy?

（4）How do you think effective publicity and promotion of rural sports policies can be carried out to increase villagers' participation and awareness?

（5)How do you think we can strengthen the cooperation and coordination among the government, social organisations, farmers and other parties in the development of rural sports?

3. Summaries.

3.1 Thanks to the interviewees for being interviewed and sharing their valuable experiences.

3.2 Summarise the main points and ideas discussed during the visit.

3.3 Express gratitude and advise of plans for later communication.
